# Dual‐Cascade Activatable Nanopotentiators Reshaping Adenosine Metabolism for Sono‐Chemodynamic‐Immunotherapy of Deep Tumors

**DOI:** 10.1002/advs.202207200

**Published:** 2023-02-02

**Authors:** Meixiao Zhan, Fengshuo Wang, Yao Liu, Jianhui Zhou, Wei Zhao, Ligong Lu, Jingchao Li, Xu He

**Affiliations:** ^1^ Guangdong Provincial Key Laboratory of Tumor Interventional Diagnosis and Treatment Zhuhai People's Hospital (Zhuhai hospital affiliated with Jinan University) Jinan University Zhuhai Guangdong 519000 China; ^2^ State Key Laboratory for Modification of Chemical Fibers and Polymer Materials College of Biological Science and Medical Engineering Donghua University Shanghai 201620 China

**Keywords:** adenosine metabolism, cancer therapy, deep tumors, immunotherapy, nanopotentiators

## Abstract

Immunotherapy is an attractive treatment strategy for cancer, while its efficiency and safety need to be improved. A dual‐cascade activatable nanopotentiator for sonodynamic therapy (SDT) and chemodynamic therapy (CDT)‐cooperated immunotherapy of deep tumors via reshaping adenosine metabolism is herein reported. This nanopotentiator (NP_MCA_) is constructed through crosslinking adenosine deaminase (ADA) with chlorin e6 (Ce6)‐conjugated manganese dioxide (MnO_2_) nanoparticles via a reactive oxygen species (ROS)‐cleavable linker. In the tumor microenvironment with ultrasound (US) irradiation, NP_MCA_ mediates CDT and SDT concurrently in deep tumors covered with 2‐cm tissues to produce abundant ROS, which results in dual‐cascade scissoring of ROS‐cleavable linkers to activate ADA within NC_MCA_ to block adenosine metabolism. Moreover, immunogenic cell death (ICD) of dying tumor cells and upregulation of the stimulator of interferon genes (STING) is triggered by the generated ROS and Mn^2+^ from NP_MCA_, respectively, leading to activation of antitumor immune response. The potency of immune response is further reinforced by reducing the accumulation of adenosine in tumor microenvironment by the activated ADA. As a result, NP_MCA_ enables CDT and SDT‐cooperated immunotherapy, showing an obviously improved therapeutic efficacy to inhibit the growths of bilateral tumors, in which the primary tumors are covered with 2‐cm tissues.

## Graphical abstract

An ultrasound and tumor microenvironment dual‐cascade activatable nanopotentiator is constructed for sono‐chemodynamic‐immunotherapy of deep tumors. The nanopotentiator produces abundant reactive oxygen species for inducing immunogenic cell death and activating adenosine deaminase to reshape the adenosine metabolism. Thus, the antitumor immune response is obviously amplified by sono‐chemodynamic‐cooperated immunotherapy to effectively treat deep 4T1 tumors covered with 2‐cm tissues.

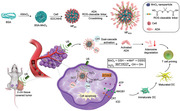

## 1 Introduction

Immunotherapy has been widely used for cancer treatment because of its advantages including strong specificity, wide applicability, and the capacity to remove residual cancer cells and prevent tumor recurrence.^[^ ^1^ ^]^ However, its therapeutic efficacy is often low as the tumors create immunosuppressive microenvironment.^[^ ^2^ ^]^ Particularly, adenosine is one of the important negative feedbacks in immunosuppressive tumor microenvironment that can weaken the immune responses.^[^ ^3^ ^]^ Adenosine is converted from adenosine triphosphate (ATP) by ectonucleotidases.^[^ ^4^ ^]^ In view of high levels of ATP during immunogenic cell death (ICD) of dying cancer cells after various treatments, adenosine will accumulate in tumor microenvironment.^[^ ^5^ ^]^ The produced adenosine can regulate the functions and proliferation of T cells, leading to the formation of regulatory T (T_reg_) cells. Therefore, inhibition of adenosine signals is highly desired to reverse the immunosuppressive tumor microenvironment for effective immunotherapy.

Different strategies have been adopted to modulate the immunosuppressive effect of adenosine, such as inhibiting ectonucleotidases activity to reduce the conversion of ATP into adenosine, blocking the binding of adenosine with T cell receptor, and degrading intracellular adenosine using adenosine deaminase (ADA).^[^ ^6^ ^]^ However, these strategies have the limitations of poor selectivity and low safety because the antagonists and enzymes show unsatisfactory accumulation in targeting tumor sites. To address the concern of uncontrolled release of immunotherapeutic agents, activatable immunotherapeutic nanoparticles that can specifically unleash cargo upon response to different stimuli have been widely developed.^[^ ^7^ ^]^ For example, a second near‐infrared (NIR‐II) photoactivatable organic polymer nanoparticle with conjugation of an A2AR antagonist has been reported to improve the efficacy and safety of photothermal‐immunotherapy.^[^ ^8^ ^]^ Nevertheless, the applications of photoactivatable nanosystems are more suitable for superficial tumors as the tissue penetration depths are limited.^[^ ^9^ ^]^

In contrast to light, ultrasound (US) can overcome the penetration obstacle as it can penetrate deeply into biological tissues, and thus has been used for sonodynamic therapy (SDT) of deep‐seated tumors.^[^ ^10^ ^]^ Moreover, US shows the advantages of good selectivity and efficient controllability, US‐responsive nanosystems have been developed for the precise delivery of immunotherapeutic agents to targeting regions for immunotherapy.^[^ ^11^ ^]^ Alternatively, tumor microenvironment‐responsive nanoplatforms that enable controlled releases of cargos upon responses to endogenous hallmarks in the tumors also do not have penetration limitations.^[^ ^12^ ^]^ However, the therapeutic efficacies are still low for these US‐ and tumor microenvironment‐responsive nanosystems due to the insufficient activation of therapeutics.^[^ ^13^ ^]^ To improve the activation efficacy, dual‐responsive nanosystems that integrate the sensitivity to both exogenous and endogenous stimuli have been reported.^[^ ^14^ ^]^ Light and tumor microenvironment dual‐responsive nanomedicines have been widely developed for cancer therapy,^[^ ^15^ ^]^ while the uses of US and tumor microenvironment dual‐responsive nanoparticles to achieve effective immunotherapy have not been explored.

**Figure 1:**
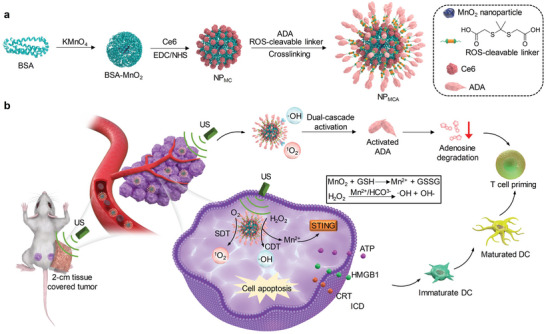
US and tumor microenvironment dual‐cascade activatable nanopotentiator (NP_MCA_) for CDT and SDT‐cooperated immunotherapy. a) Schematic illustration of the fabrication of NP_MCA_. b) Schematic illustration of the activation mechanism, ICD induction, and immune response activation mediated by NP_MCA_ for CDT and SDT‐cooperated immunotherapy.

We herein report a US and tumor microenvironment dual‐cascade activatable nanopotentiator for reshaping adenosine metabolism and combinational immunotherapy of cancer. Such a nanopotentiator (NP_MCA_) contains chlorin e6 (Ce6)‐conjugated manganese dioxide (MnO_2_) nanoparticles and ADA, which are crosslinked by a reactive oxygen species (ROS)‐cleavable linker (**Figure** **1**a). Ce6 was chemically conjugated onto MnO_2_ nanoparticles to avoid the unwanted release in blood circulation. Ce6 acts as a sonosensitizer to produce singlet oxygen (^1^O_2_) and mediate SDT under US irradiation. MnO_2_ nanoparticles react with endogenous glutathione (GSH) in tumor microenvironment to produce Mn^2+^, and Mn^2+^ can mediate the conversion of hydrogen peroxide (H_2_O_2_) into hydroxyl radical (·OH) for chemodynamic therapy (CDT). The abundant ROS produced by the combinational action of SDT and CDT not only induces ICD of dying cancer cells, but also scissors ROS‐cleavable linkers for dual‐cascade activation of ADA (Figure 1b). Moreover, the released Mn^2+^ from NP_MCA_ can further upregulate the activity of the stimulator of interferon genes (STING).^[^ ^16^ ^]^ In view of the ICD effect, STING activation, and adenosine consumption by the activated ADA, the antitumor immune response is obviously amplified. Therefore, NP_MCA_‐mediated CDT and SDT‐cooperated immunotherapy can obviously suppress the growths of deep 4T1 tumors covered with 2‐cm tissues.

## 2 Results and Discussion

### 2.1 Screening of Sonosensitizers

**Figure 2:**
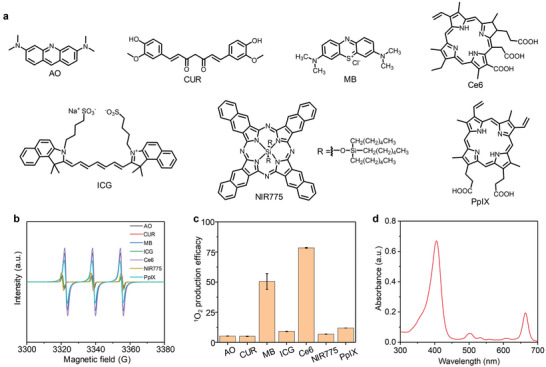
Screening of sonosensitizers. a) Chemical structures of different sonosensitizers including AO, CUR, MB, Ce6, ICG, NIR775, and PpIX. b) ESR measurements of ^1^O_2_ generation for different sonosensitizers at the same concentration under US treatment. c) The ^1^O_2_ production efficacy of the small‐molecule sonosensitizers under US treatment (n = 3). d) UV–vis spectrum of Ce6. The data are presented as the means ± SDs.

To synthesize nanopotentiators with good SDT effect, sonodynamic ^1^O_2_ generation property of different small‐molecule sonosensitizers was investigated using electron spin resonance (ESR). The common small‐molecule sonosensitizers include acridine orange (AO), curcumin (CUR), methylene blue (MB), Ce6, indocyanine green (ICG), silicon 2,3‐naphthalocyanine bis(trihexylsilyloxide) (NIR775) and protoporphyrin IX (PpIX) (**Figure** **2**a). ESR results showed that all the sonosensitizers could produce ^1^O_2_ under US treatment, and the ESR intensity for Ce6 was much higher than those for other sonosensitizers (Figure 2b), suggesting the highest sonodynamic ^1^O_2_ generation efficacy of Ce6. The ^1^O_2_ generation efficacy of Ce6 was 1.6‐fold higher than that of MB, and at least 6.6‐fold higher relative to those of the other sonosensitizers (Figure 2c). UV–vis spectrum showed that Ce6 had obvious optical absorbance in the range of 300–700 nm (Figure 2d), which should contribute to its excellent ^1^O_2_ generation under US treatment. Therefore, Ce6 was selected as the optimized sonosensitizer to synthesize nanopotentiators.

### 2.2 Fabrication and Characterization of Nanoparticles

**Figure 3:**
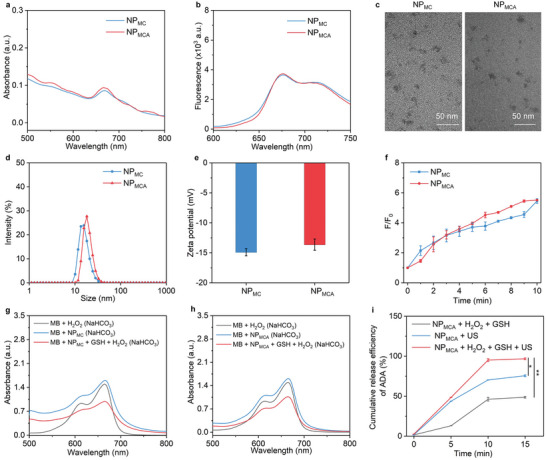
Fabrication and characterization of nanoparticles. a) UV–vis absorbance spectra of NP_MC_ and NP_MCA_. b) Fluorescence spectra of NP_MC_ and NP_MCA_. c) TEM images of NP_MC_ and NP_MCA_. d) Profiles of hydrodynamic size of NP_MC_ and NP_MCA_. e) Measurement of zeta potential of NP_MC_ and NP_MCA_ (n = 3). f) Fluorescence enhancement (F/F_0_) for SOSG solutions containing NP_MC_ and NP_MCA_ under US treatment for different times (n = 3). g) Absorbance spectra of MB solutions containing NP_MC_ and/or H_2_O_2_. h) Absorbance spectra of methylene blue (MB)MB solutions containing NP_MCA_ and/or H_2_O_2_. i) Cumulative release curves of ADA for NP_MCA_ solutions without or with the addition of H_2_O_2_ under US treatment (n = 3). The data are presented as the means ± SDs. The p values are calculated using two‐tailed unpaired t test, *p < 0.05, **p < 0.01.

NP_MC_ was synthesized by conjugating sonosensitizer Ce6 onto bovine serum albumin (BSA)‐MnO_2_ nanoparticles. The characteristic peak of Ce6 at 664 nm could be detected in the absorbance spectrum of NP_MC_ (**Figure** **3**a), which however was not observed in that of BSA‐MnO_2_ nanoparticles (Figure S1a, Supporting Information), confirming the successful synthesis of NP_MC_. The hydrodynamic size and zeta potential of BSA‐MnO_2_ nanoparticles were measured to be 10.0 nm and −13.8 mV, respectively (Figure S1b,c, Supporting Information). Via crosslinking of NP_MC_ with ADA using ROS‐cleavable linkers, NP_MCA_ were fabricated. The loading ratio of ADA within NP_MCA_ was calculated to be 13.4%. As shown in the absorbance spectrum of NP_MC_, the characteristic peak of Ce6 at 664 nm was similarly observed. Due to the presence of Ce6, NP_MC_ and NP_MCA_ similarly displayed distinct fluorescence signals ranging from 650–750 nm (Figure 3b), while BSA‐MnO_2_ nanoparticles did not have any fluorescence properties (Figure S1d, Supporting Information). These results verified that the crosslinking of ADA did not affect the absorbance and fluorescence properties of nanoparticles. Transmission electron microscope (TEM) images showed that both NP_MC_ and NP_MCA_ had a spherical morphology, and they were well dispersed without obvious aggregation (Figure 3c), but the dimension of NP_MCA_ was larger than that of NP_MC_. The hydrodynamic size was measured to be 15.7 nm for NP_MC_ and 18.2 nm for NP_MCA_ (Figure 3d). Both NP_MC_ and NP_MCA_ showed good stability when they were dispersed in water, phosphate‐buffered saline (PBS), and cell culture medium (Figure S2, Supporting Information). The zeta potential of NP_MCA_ (−13.6 mV) was similar to that of NP_MC_ (−14.9 mV) due to their coincident surface components (Figure 3e). Hemolysis assay showed that the hemolysis ratios of blood red cells were less than 5.0% after incubation with NP_MC_ and NP_MCA_ at the Ce6 concentration of 3.2–50 µg mL^−1^ (Figure S3, Supporting Information), indicating the negligible hemolysis effect of both nanoparticles.

The sonodynamic and chemodynamic properties of nanoparticles were then evaluated. Sonodynamic ^1^O_2_ generation was confirmed by measuring the fluorescence intensity of ^1^O_2_ probe (SOSG). The fluorescence intensity of SOSG was gradually increased under US treatment for both NP_MC_ and NP_MCA_ solutions (Figure S4, Supporting Information). The fluorescence enhancement (F/F_0_) similarly reached 5.8 for NP_MC_ and NP_MCA_ after US treatment for 10 min (Figure 3f). These results confirmed the effective generation of ^1^O_2_ for NP_MC_ and NP_MCA_, and their sonodynamic ^1^O_2_ generating efficacies were almost consistent. By using MB as ·OH indicator, the absorbance of MB solution containing NP_MC_ and H_2_O_2_ (100 µm) was reduced compared to that of MB + H_2_O_2_ and MB + NP_MC_ group (Figure 3g). Weaker absorbance could also be observed for MB when it was mixed with NP_MCA_ and H_2_O_2_ (100 µm) than that in MB + NP_MCA_ group (Figure 3h). These results verified the production of ·OH by NP_MC_ and NP_MCA_ in the presence of H_2_O_2_.

The dual‐cascade activation of NP_MCA_ was then evaluated by measuring the release amount of fluorescein isothiocyanate (FITC)‐conjugated ADA from nanoparticles. For NP_MCA_ without the addition of H_2_O_2_ and US treatment, the release of ADA was negligible (Figure 3i). The release of ADA could be observed after incubation of NP_MCA_ with H_2_O_2_, and higher release efficacy of ADA was observed after US treatment of NP_MCA_. In contrast, ADA release efficacy after US treatment and H_2_O_2_ incubation was higher than that in NP_MCA_ + H_2_O_2_ and NP_MCA_ + US groups. These results verified that US and H_2_O_2_ could synergistically promote the ADA release from NP_MCA_. This should be attributed to the cleavage of ROS‐cleavable linkers by the generated ^1^O_2_ and ·OH for dual‐cascade activation of NP_MCA_.

### 2.3 In Vitro Therapeutic Efficacy and ICD Evaluation

**Figure 4:**
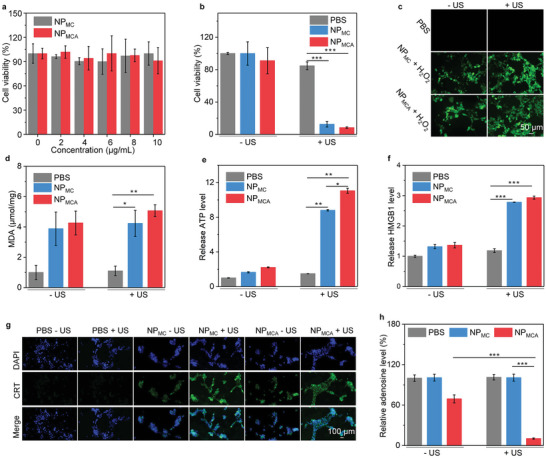
In vitro therapeutic efficacy and ICD analysis. a) Cell viability analysis of NP_MC_‐ and NP_MCA_‐treated cells at different Ce6 concentrations for 24 h (n = 5). b) Cell viability analysis of 4T1 cells in PBS‐, NP_MC_‐ and NP_MCA_‐treated groups without or with US treatment (n = 5). c) Fluorescence images of 4T1 cells in PBS‐, NP_MC_‐ and NP_MCA_‐treated groups without or with US treatment in the presence of ROS probes. d) MDA levels for 4T1 cells in PBS‐, NP_MC_‐ and NP_MCA_‐treated groups without or with US treatment (n = 5). e) Released ATP levels for 4T1 cells in various treatment groups (n = 5). f) Extracellular HMGB1 levels for 4T1 cells in PBS‐, NP_MC_‐ and NP_MCA_‐treated groups without or with US treatment (n = 5). g) Immunofluorescence CRT staining images of 4T1 cells after different treatments. h) Intracellular adenosine levels for 4T1 cells after different treatments (n = 5). The data are presented as the means ± SDs. The p values are calculated using two‐tailed unpaired t test, *p < 0.05, **p < 0.01, and ***p < 0.001.

In view of the fluorescence property of Ce6, the cellular uptake of NP_MC_ and NP_MCA_ by cancer cells was first evaluated using flow cytometry. After incubation of cancer cells with NP_MC_ or NP_MCA_, the fluorescence intensity of cancer cells remarkably increased compared to that of PBS control cells (Figure S5, Supporting Information), verifying the cellular endocytosis effect. The cytotoxicity of NP_MC_ and NP_MCA_ was investigated to confirm their biocompatibility for biomedical applications. The cell viability was nearly 100% after incubation with NP_MC_ and NP_MCA_ at the studied Ce6 concentration range for 24 h (**Figure** **4**a), suggesting the negligible cytotoxicity of NP_MC_ and NP_MCA_. Without US treatment, the cell viability did not have an obvious decrease after incubation with NP_MC_ and NP_MCA_ (Figure 4b). In contrast, the cell viability was significantly reduced for NP_MC_‐ and NP_MCA_‐treated cells with US treatment, verifying the cell‐killing effect via the combinational action of SDT and CDT.

Fluorescence imaging of cells was conducted to confirm intracellular ROS generation using the ROS probe (H_2_DCFDA). No green fluorescence signals could be observed for PBS control cells, while green fluorescence signals were found in NP_MC_ + H_2_O_2_ and NP_MCA_ + H_2_O_2_ cells, which should be due to the generation of ·OH via chemodynamic effect (Figure 4c). In NP_MC_ + H_2_O_2_ + US and NP_MCA_ + H_2_O_2_ + US cells, the green fluorescence signals were much stronger than those in NP_MC_ + H_2_O_2_ and NP_MCA_ + H_2_O_2_ cells. The enhanced ROS generation should be attributed to the total ^1^O_2_ and ·OH generation through sonodynamic and chemodynamic effect, respectively. The lipid peroxidation of 4T1 cells was then evaluated using malondialdehyde (MDA) assay. Regardless of US treatment, the MDA levels in NP_MC_‐ and NP_MCA_‐treated cells were much higher than those in PBS control group (Figure 4d). These results confirmed the cellular lipid peroxidation because of the chemodynamic effect of MnO_2_ nanoparticles.

Extracellular ATP levels were found to increase by 8.8‐ and 11.1‐fold in NP_MC_ + US and NP_MCA_ + US groups compared to that in PBS control group, respectively (Figure 4e). Furthermore, the extracellular high‐mobility group box 1 (HMGB1) levels were investigated using ELISA kit. After treatment of NP_MC_ and NP_MCA_ with US treatment, the extracellular HMGB1 levels were increased by around 2.9‐fold compared to that in control group (Figure 4f), which were slightly increased for NP_MC_ and NP_MCA_ treatments in the presence of H_2_O_2_. Immunofluorescence calreticulin (CRT) staining images showed that the green fluorescence signals in NP_MC_ + H_2_O_2_ + US and NP_MCA_ + H_2_O_2_ + US groups were obviously observed, which however were hardly detected in NP_MC_ + H_2_O_2_ and NP_MCA_ + H_2_O_2_ groups (Figure 4g). These results suggested that the CRT levels in these two groups were remarkably upregulated. Overall, both NP_MC_ and NP_MCA_ with US treatment could effectively induce ICD via upregulating the levels of ATP, CRT, and HMGB1.

To verify the role of activated ADA, the intracullular adenosine levels for cancer cells after treatments were evaluated using high‐performance liquid chromatography (HPLC). Only with H_2_O_2_ activation, the adenosine level in NP_MCA_ – US group was 1.4‐fold lower than that in PBS control group, while in NP_MCA_ + US group obviously reduced by around 9.7‐fold compared to those in the other groups (Figure 4h). These results suggested that NP_MCA_ could be activated to release ADA for the effective consumption of adenosine.

### 2.4 Tumor Growth Inhibition Evaluation

**Figure 5:**
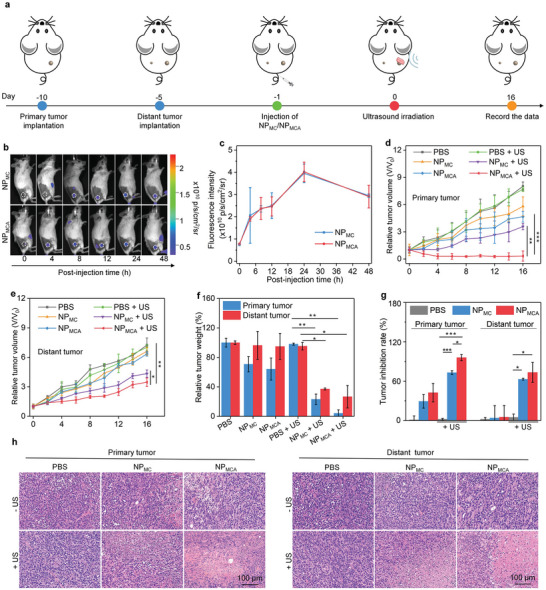
Tumor growth inhibition evaluation. a) Schematic of the establishment of bilateral tumor model, nanoparticle injection, and chicken breast tissue coverage and US treatment of primary tumors. b) Fluorescence images of mice in NP_MC_‐ and NP_MCA_‐injected groups (the tumor regions were indicated by white dotted circles). c) Fluorescence intensity of tumor sites in NP_MC_‐ and NP_MCA_‐injected mice at different times (n = 3). d) Relative tumor volume of chicken breast tissue‐covered 4T1 primary tumors in PBS‐, NP_MC_‐ and NP_MCA_‐treated mice without or with US treatment (n = 5). e) Relative tumor volume of 4T1 distant tumors in PBS‐, NP_MC_‐ and NP_MCA_‐treated mice without or with US treatment (n = 5). f) Total weight of primary and distant tumors in PBS‐, NP_MC_‐ and NP_MCA_‐treated mice without or with US treatment (n = 5). g) Tumor inhibition efficacy analysis (n = 5). h) Images of H&E stained primary and distant tumors in PBS‐, NP_MC_‐ and NP_MCA_‐treated mice without or with US treatment. The data are presented as the means ± SDs. The p values are calculated using two‐tailed unpaired t test, *p < 0.05, **p < 0.01, and ***p < 0.001.

Bilateral 4T1 tumor‐bearing mouse models were used to investigate the deep‐tissue therapeutic efficacy. At 24 h after intravenous injection of NP_MC_ and NP_MCA_, the primary tumors were covered with 2‐cm chicken breast tissues and then treated with US for 10 min (**Figure** **5**a). To optimize timepoints of US treatment for cancer therapy, the accumulation of nanoparticles in tumor tissues was investigated. As shown in the fluorescence images of mice, the tumors showed fluorescence signals after injection of NP_MC_ and NP_MCA_, and the signals gradually increased until 24 h, and then declined (Figure 5b). In addition, the fluorescence intensity for tumors of NP_MC_‐ and NP_MCA_‐injected mice were almost consistent at the same post‐injection timepoints. The highest fluorescence intensity of tumors for NP_MC_‐ and NP_MCA_‐injected mice was observed at 24 h post‐injection timepoint (Figure 5c). These results suggested that both NP_MC_ and NP_MCA_ could effectively accumulate into tumor sites, and they showed the highest accumulation at 24 h. Such a high tumor accumulation efficacy of NP_MC_ and NP_MCA_ should be attributed to their small sizes and excellent stability. Bio‐distribution analysis showed that both NP_MC_ and NP_MCA_ had a high accumulation in tumor, kidney, and liver, while limited accumulation in lung, heart, and spleen (Figure S6, Supporting Information). Quantitative analysis of Mn element in different tissues also showed that MnO_2_ nanoparticles had a similar accumulation in tumor, kidney, and liver (Figure S7, Supporting Information).

For primary tumors, the growth in NP_MC_‐ and NP_MCA_‐injected mice without US treatment was slightly inhibited compared to that in PBS control group (Figure 5d), which should be due to the therapeutic efficacy of CDT. In contrast, the tumor growths in NP_MC_‐ and NP_MCA_‐injected and US‐treated mice were remarkably inhibited. The treatment of NP_MC_ and NP_MCA_ without US treatment did not remarkably inhibit the distant tumor growths in mice, and the tumor growths were similar to that for PBS control (Figure 5e). However, the growth of distant tumors was obviously inhibited in NP_MC_ + US and NP_MCA_ + US groups. The tumor weight in each group was then measured, which in NP_MC_ + US and NP_MCA_ + US groups was much lower than those in the other groups (Figure 5f). Moreover, the total weight in NP_MCA_ + US group (0.05 g) was 5.8‐fold lower than that in NP_MC_ + US group (0.29 g). The tumor inhibitory efficacy for primary tumors in NP_MCA_ + US group was 95.8%, which was 1.3‐fold higher relative to that in NP_MC_ + US group (72.9%) (Figure 5g). The tumor inhibitory efficacy for distant tumors was 73.3% for NP_MC_ + US and 62.8% for NP_MCA_ + US group, respectively. These results suggested that both NP_MC_ and NP_MCA_ with US treatment showed an obvious tumor growth inhibitory effect and the antitumor ability of NP_MCA_ was higher than that of NP_MC_.

Histological staining of tumor tissues was conducted to further evaluate the therapeutic efficacy. Cell damage was observed for the primary tumors in NP_MC_ – US, NP_MCA_ –US, NP_MC_ + US, and NP_MCA_ + US groups, while it was only detected for the distant tumors in NP_MC_ + US, and NP_MCA_ + US groups (Figure 5h). The severest cell damage in primary and distant tumors was observed in NP_MCA_ + US group. These results further verified that NP_MCA_ with US treatment showed the best tumor cell‐killing efficacy.

### 2.5 Intratumoral Lipid Peroxidation and ICD Evaluation

**Figure 6:**
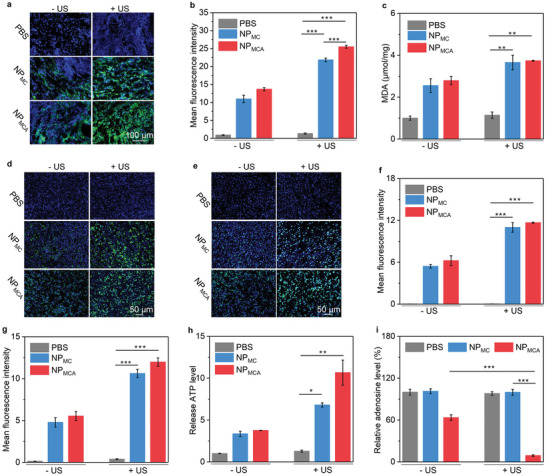
Intratumoral lipid peroxidation and ICD evaluation. a) Fluorescence images of produced ROS in chicken breast tissue‐covered primary tumors in PBS‐, NP_MC_‐ and NP_MCA_‐treated mice without or with US treatment. b) Mean fluorescence intensity of the produced ROS in chicken breast tissue‐covered primary tumors (n = 5). c) Lipid peroxidation assay of chicken breast tissue‐covered primary tumors in these treatment groups (n = 5). d) Immunofluorescence CRT staining images of chicken breast tissue‐covered primary tumors. e) Immunofluorescence HMGB1 staining images of chicken breast tissue‐covered primary tumors. f) Relative fluorescence intensity of CRT in tumors after different treatments (n = 5). g) Relative fluorescence intensity of HMGB1 in tumors after different treatments (n = 5). h) Relative ATP levels in chicken breast tissue‐covered primary tumors in these treatment groups (n = 5). i) Relative adenosine levels in chicken breast tissue‐covered primary tumors (n = 5). The data are presented as the means ± SDs. The p values are calculated using two‐tailed unpaired t test, *p < 0.05, **p < 0.01, and ***p < 0.001.

The ROS generation in chicken breast tissue‐covered tumor tissues and lipid peroxidation was then evaluated. Compared to PBS control group in which nearly no green fluorescence signals were detected in primary tumors, weak fluorescence signals could be detected in the tumors for NP_MC_‐ and NP_MCA_‐treated mice without US treatment (**Figure** **6**a). The ROS generation in these two groups was attributed to the formation of ·OH via chemodynamic reaction. The green fluorescence signals in NP_MC_ + US and NP_MCA_ + US groups were similar, and they were much stronger than those in the other groups, which should be due to the generation of ·OH and ^1^O_2_ via chemodynamic and sonodynamic effects concurrently. The fluorescence intensities of green signals in NP_MC_ + H_2_O_2_ + US and NP_MCA_ + H_2_O_2_ + US groups were around 25‐fold higher than those in NP_MC_ + H_2_O_2_ and NP_MCA_ + H_2_O_2_ groups (Figure 6b). The lipid peroxidation levels of tumors after treatments were also investigated. After treatment with NP_MC_ and NP_MCA_ regardless of US treatment, the lipid peroxidation levels in tumor tissues were significantly increased (Figure 6c). This verified the CDT effect of NP_MC_ and NP_MCA_ for tumor ablation.

To evaluate the ICD effect, the ATP release and CRT and HMGB1 expression levels in primary tumors covered with 2‐cm chicken breast tissues were studied. Immunofluorescence CRT staining signals were detected in NP_MC_ and NP_MCA_ injection groups regardless of US treatment, but not in PBS control group (Figure 6d). The fluorescence staining signals in NP_MC_ + US and NP_MCA_ + US groups were stronger than those in NP_MC_ – US and NP_MCA_ – US groups. The treatments of NP_MC_ and NP_MCA_ also upregulated HMGB1 levels, and the HMGB1 staining signals in NP_MC_ + US and NP_MCA_ + US groups were further enhanced as compared to those in NP_MC_ – US and NP_MCA_ – US groups (Figure 6e). The quantitative data suggested that the fluorescence intensity of CRT in NP_MC_ + US and NP_MCA_ + US groups increased by 10.9‐ and 11.6‐fold compared with PBS group, respectively (Figure 6f). The fluorescence intensity of HMGB1 in NP_MC_ + US and NP_MCA_ + US groups increased by 10.6‐ and 11.9‐fold (Figure 6g). NP_MC_ and NP_MCA_ injection without US treatment increased the intratumoral ATP level by 3.3‐ and 3.7‐fold, respectively, but the NP_MC_ and NP_MCA_ injection with US treatment significantly increased the ATP level by 6.8‐ and 10.6‐fold (Figure 6h). The ATP level in tumors for NP_MCA_ + US group was higher than that for NP_MC_ + US group, which may be because the interference of adenosine metabolism affected the intratumoral accumulation of ATP. These results suggested that NP_MC_ and NP_MCA_ injection with US treatment could effectively induce ICD effect in deep tumors. The adenosine level in tumor tissues was slightly reduced by 1.6‐fold in NP_MCA_ – US group compared to that in PBS control group, while it was reduced by 11.0‐fold in NP_MCA_ + US group (Figure 6i). These results confirmed the activation of ADA in tumor tissues for adenosine consumption.

Although the solid tumors showed hypoxic microenvironment, the existing oxygen could be utilized to produce ROS via SDT effect and thus effectively activated ADA with the synergistic action of CDT. The combination of SDT and CDT could overcome the issue of tumor hypoxia, thus showing improved efficacy in activating ADA and inducing ICD. As immunotherapy played the dominant role in treating tumors, NP_MCA_ showed a much higher antitumor efficacy than NP_MC_.

### 2.6 Evaluation of DC and T Cell Activation

**Figure 7:**
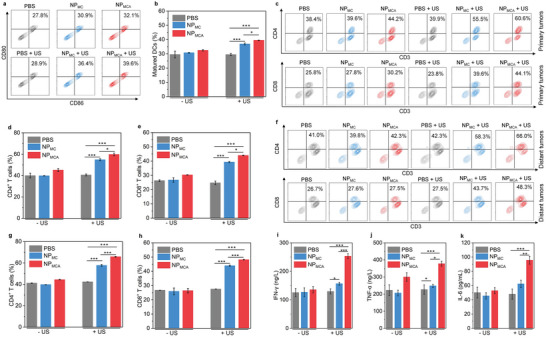
In vivo immune response evaluation. a) Flow cytometry analysis of DCs in lymphatic nodules of mice after different treatments. b) Number of matured DCs in tumor‐draining lymph nodes in PBS‐, NP_MC_‐ and NP_MCA_‐treated mice without or with US treatment (n = 5). c) Flow cytometry analysis of CD4+ and CD8+ T cells in primary tumors of mice after different treatments. d) Number of CD4^+^ T cells in chicken breast tissue‐covered primary tumors in PBS‐, NP_MC_‐ and NP_MCA_‐treated mice without or with US treatment (n = 5). e) Number of CD8^+^ T cells in chicken breast tissue‐covered primary tumors (n = 5). f) Flow cytometry analysis of CD4+ and CD8+ T cells in distant tumors of mice after different treatments. g) Number of CD4^+^ T cells in distant tumors in PBS‐, NP_MC_‐ and NP_MCA_‐treated mice without or with US treatment (n = 5). h) Number of CD8^+^ T cells in distant primary tumors (n = 5). Serum levels of i) IFN‐*γ*, j) TNF‐*α*, and k) IL‐6 in PBS‐, NP_MC_‐, and NP_MCA_‐treated mice without or with US treatment (n = 5). The data are presented as the means ± SDs. The p values are calculated using two‐tailed unpaired t test, *p < 0.05, **p < 0.01, and ***p < 0.001.

The dendritic cells (DCs) in tumor‐draining lymph nodes were evaluated. Compared to the numbers of matured DCs in PBS control groups, which overall increased after the treatment of NP_MC_ and NP_MCA_ regardless of US treatment (**Figure** **7**a). The number of matured DCs in NP_MC_ + US (36.9%) and NP_MCA_ + US (39.5%) groups was higher than that in NP_MC_ – US (29.4%) and NP_MCA_ – US (32.4%) groups (Figure 7b). These results suggested that treatment of NP_MC_ and NP_MCA_ with US treatment could greatly promote the maturation of DCs, which should be attributed to the combinational action of STING activation by Mn^2+^ and the ICD effect caused by CDT and SDT.

To confirm the activation of antitumor immune response, effector T cells in tumors were then analyzed. In chicken breast tissues‐covered primary tumors, increased populations of CD4^+^ T cells could be detected in NP_MC_‐ and NP_MCA_‐injected mice with US treatment compared to that in the other groups (Figure 7c). The number of CD4^+^ T cells in NP_MC_ + US and NP_MCA_ + US groups was 54.8% and 59.7%, respectively, which was at least 1.5‐fold higher than that for PBS control and the other treatments (Figure 7d). In distant tumors, the highest number for CD4^+^ T cells was also observed in NP_MCA_ + US group (65.8%), 1.1‐fold higher than that in NP_MC_ + US group (57.7%), and at least 1.6‐fold higher than that in the other groups (Figure 7f,g). The number of CD8^+^ T cells in chicken breast tissues‐covered primary tumors was measured to be 26.2% for PBS control, 26.7% for NP_MC_ – US, 30.3% for NP_MCA_ – US, 39.3% for NP_MC_ + US, and 43.9% for NP_MCA_ + US group, respectively (Figure 7e). As for CD8^+^ T cells in distant tumors, the number increased to 48.1% after NP_MCA_ injection with US treatment compared to 26.7% in PBS control group (Figure 7h), which was also higher than that in NP_MC_ – US (25.9%), NP_MCA_ – US (26.4%), and NP_MC_ + US (43.9%) group. These results verified that NP_MCA_ with US treatment showed the highest efficacy in promoting the activation of immune T cells. The amplified immunological effect of NP_MCA_ over NP_MC_ should be due to the role of ADA activation.

Some immune‐related cytokines can promote DC maturation, antigen presentation, and T cell proliferation, thus playing key roles in antitumor immune response.^[^ ^17^ ^]^ The serum levels of cytokines were evaluated to further confirm the activation of immune response. The highest levels of interferon‐γ (IFN‐γ) were observed in NP_MCA_ + US group, which was 1.6‐ and 2.0‐fold higher than that in NP_MC_ + US and control group, respectively (Figure 7i). The serum level of tumor necrosis factor‐α (TNF‐α) in NP_MCA_ + US group was increased by 1.7‐fold, while in NP_MC_ + US group was only increased by 1.3‐fold (Figure 7j). The NP_MCA_ with US treatment also increased the level of interleukin‐6 (IL‐6) in serum by 1.9‐fold, which was much obvious than the other treatments (Figure 7k). Therefore, the treatment of NP_MCA_ with US treatment could effectively promote the secretion of cytokines for effective immunotherapy.

### 2.7 Biosafety Evaluation

All mice after different treatments showed unchanged body weights during monitoring period (Figure S8, Supporting Information). The histological morphologies of kidney, spleen, and heart for mice after NP_MCA_ injection and US treatment did not have any changes and were similar to those in PBS control group (Figure S9, Supporting Information). These results confirmed the good biosafety of NP_MCA_ for cancer treatment.

## 3 Conclusion

We have reported a nanopotentiator (NP_MCA_) that can be activated by tumor microenvironment and US to mediate SDT, CDT, and inhibition of adenosine metabolism for enhanced immunotherapy of deep tumors. NP_MCA_ could specifically release ADA via scissoring ROS‐cleavable linkers by the generated ·OH and ^1^O_2_ through CDT and SDT effect upon US treatment in the presence of H_2_O_2_ in tumor microenvironment, which leads to interference of adenosine metabolism. Due to the excellent tissue penetrating capability of US, NP_MCA_ was able to mediate the ROS generation in deep tumors covered with 2‐cm chicken breast tissues. In addition to direct killing of tumor cells, these generated ROS also induced ICD of dying tumor cells, which played an important role in triggering the maturation of DCs and priming of T cells. Thus, the populations of DCs and CD4^+^ and CD8^+^ T cells in NP_MCA_ injected and US‐treated group were increased. Through combining CDT, SDT, and immunotherapy, NP_MCA_ achieved effective inhibition of growths of deep tumors. This study presents the first tumor microenvironment and US dual‐cascade activatable nanoplatform for effective treatment of deep tumors. Because of the excellent tissue penetration depth of this therapeutic strategy, its possible to be used for the treatment of orthotopic tumor models (such as hepatic carcinoma and pancreatic cancer) in deep tissues will be explored.

## 4 Experimental Section

### Synthesis of BSA‐Stabilized MnO_2_ (BSA‐MnO_2_) Nanoparticles

KMnO_4_ (32 mg) and BSA (250 mg) were dissolved in 10 mL water, and the obtained solution was reacted under stirring for 3 h. The solution was then dialyzed using dialysis membranes at 25 °C for 12 times. After further ultrafiltration, BSA‐MnO_2_ nanoparticles were obtained.

### Synthesis of Ce6‐Conjugated BSA‐MnO_2_ Nanoparticles (NP_MC_)

Ce6 (1 mg) dissolved in 5 mL dimethyl sulfoxide (DMSO) was reacted with hydrochloride crystalline (EDC, 2 mg) and N‐hydroxysuccin‐imide (NHS, 2 mg) at 25 °C under the dark for 3 h to activate the carboxyl groups. The activated Ce6 was then mixed with BSA‐MnO_2_ nanoparticles, and the reaction was continued at 25 °C under the dark for 72 h. The solution was dialyzed at 25 °C for 12 times to remove the raw materials. After ultrafiltration, the products (NP_MC_) were obtained.

### Synthesis of ROS‐Cleavable Linker

ROS‐cleavable linker with terminal carboxyl groups on each side was synthesized according to previous work.^[^ ^7c^ ^]^

### Synthesis of NP_MCA_

ROS‐cleavable linker (10 mg), EDC (40 mg), and NHS (22 mg) were co‐dissolved in 0.1 mL DMSO and the reaction was continued at 25 °C for 3 h to activate the carboxyl groups. The activated ROS‐cleavable linkers were then mixed with ADA and NP_MC_ in 2 mL PBS. The reaction was continued at 4 °C under the dark for 24 h, and the obtained solution was purified via ultrafiltration (molecular weight cut‐off = 30 kDa) at 4 °C to obtain NP_MCA_.

### Evaluation of Sonodynamic ^1^O_2_ Generation Efficacy

PBS solution of NP_MC_ or NP_MCA_ (1 mL) was mixed with SOSG solution (1 µL), and the formed solutions were treated with US (1.0 W cm^−2^, 50% duty cycle). The fluorescence intensities of SOSG for solutions without or with US treatments were measured using fluorescence spectrophotometer to calculate sonodynamic ^1^O_2_ generation.

### Evaluation of ·OH Generation Efficacy

PBS solution of NP_MC_ or NP_MCA_ (0.3 mL) was mixed with MB (3 mL), and H_2_O_2_ was added into the solutions. The solutions were incubated at 25 °C for 30 min. The absorbance of TMB for solutions was measured to evaluate the ·OH generation.

### Evaluation of Activatable ADA Release

To evaluate activatable ADA release, FITC‐conjugated ADA was used to fabricate FITC‐NP_MCA_. PBS solutions of FITC‐NP_MCA_ without or with the addition of H_2_O_2_ (100 µm) were treated with US (1.0 W cm^−2^, 50% duty cycle) for 10 min. The solutions were then ultrafiltrated (molecular weight cut‐off = 50 kDa) to collect lower solutions. The concentrations of FITC‐conjugated ADA in the lower solutions were measured using fluorescence spectrometer to calculate release percentages.

### Hemolysis Assay

Mouse blood red cells were incubated with PBS solutions of NP_MC_ or NP_MCA_ at different Ce6 concentrations at 25 °C for 2 h. Hemolysis assay was then conducted by measuring the absorbance of supernatants after centrifugation to precipitate the blood red cells.

### Cytocompatibility Assay

4T1 cells were incubated with NP_MC_ or NP_MCA_ at different Ce6 concentrations for 24 h. The cells were then cultured in cell culture medium containing cell counting kit‐8 (CCK‐8) for 2 h, and then the cell viability was evaluated using CCK‐8 assay.

### In Vitro Therapeutic Efficacy Evaluation

4T1 cells were incubated with NP_MC_ or NP_MCA_ at different Ce6 concentrations and H_2_O_2_ at the final concentration of 100 µm for 24 h. The cells were then treated with US (1.0 W cm^−2^, 50% duty cycle) for 3 min. After further culture in cell culture medium containing CCK‐8 for 2 h, CCK‐8 assay was used to evaluate the cell viability.

### Intracellular ROS Level Evaluation

4T1 cells were incubated with H_2_DCFDA (10 µm), NP_MC_, or NP_MCA_ at different Ce6 concentrations and H_2_O_2_ at the final concentration of 100 µm. The cells were then treated with US (1.0 W cm^−2^, 50% duty cycle) for 3 min. After that, the fluorescence images were captured to analyze intracellular ROS levels.

### Cellular Lipid Peroxidation Evaluation

4T1 cells were incubated with PBS, NP_MC_ or NP_MCA_, and H_2_O_2_ (100 µm). The cells were treated with US (1.0 W cm^−2^, 50% duty cycle) for 3 min. The treated cells were collected for lipid peroxidation assay using MDA kit according to the standard procedures.

### In Vitro ICD Induction

4T1 cells incubated in cell culture medium containing NP_MC_ or NP_MCA_ at different Ce6 concentrations and H_2_O_2_ were treated by US (1.0 W cm^−2^, 50% duty cycle, 3 min). The cells were used for the analysis of HMGB1 levels, ATP contents, and CRT expression levels.

### Tumor Model Establishment

Animal experiments were conducted according to the procedures permitted by the Institutional Anima Care and Treatment Committee of Jinan University. Bilateral mouse 4T1 tumor models were established by subcutaneously implanting 4T1 cells into the two flanks of mice (BALB/c, female, 5–7 weeks).

### Tumor Accumulation and Bio‐Distribution Analysis

A fluorescence imaging system was adopted to investigate the tumor accumulation and bio‐distribution after intravenous injection of nanoparticles.

### Intratumoral ROS Level Evaluation

At 24 h post‐injection of PBS, NP_MC_, or NP_MCA_ at the Ce6 concentration of 50 µg mL^−1^ (0.2 mL for each mouse), the primary tumors were directly injected with H_2_DCFDA and then covered with chicken breast tissues at the thickness of 2 cm. After 0.5 h, the primary tumors were treated with US (1.0 W cm^−2^, 50% duty cycle). The mice were then euthanized to extract tumors, and sections of tumor tissues were prepared for fluorescence imaging.

### Intratumoral Lipid Peroxidation Evaluation

After treatments, the primary tumors were extracted from mice for lipid peroxidation assay using MDA kit.

### Tumor Inhibitory Efficacy Evaluation

At 24 h post‐injection timepoint, the primary tumors were covered with chicken breast tissues at the thickness of 2 cm. Then the primary tumors were treated with US (1.0 W cm^−2^, 50% duty cycle) for 3 min. The tumor lengths and widths were measured to calculate the tumor volumes. Histological analysis was performed by staining tumor sections and capturing stained images using a fluorescence microscope.

### In Vivo ICD Induction

The evaluations of ATP, CRT, and HMGB1 levels in tumor tissues were conducted according to previous work.^[^ ^7d^ ^]^

### Adenosine Level Measurement

After the treatments of 4T1 cells and primary tumors, the samples were collected for the measurement of adenosine levels by HPLC.

### Evaluation of Antitumor Immune Response

The primary tumors were covered with 2‐cm chicken breast tissues and then treated with US for 10 min. After 10 days of treatments, the mice were euthanized to extract primary and distant tumors and tumor‐draining lymph nodes. These tissues were used to prepare single cell suspensions by grinding and filtering. The collected single cells were stained with antibodies, and then analyzed using a CytoFLEX flow cytometer. The tumor tissues were homogenized in PBS solution and the formed suspensions were filtered via cell strainers to obtain the single cell suspensions. The cells were stained with antibodies and then analyzed using a CytoFLEX flow cytometer.

### Cytokine Level Evaluation

After 7 days of treatment as described above, blood was collected from the mice and serum was obtained by centrifuging the blood. The serum levels of TNF‐*α*, IFN‐*γ*, and IL‐6 were measured.

### In Vivo Biosafety Evaluation

Body weights of 4T1 tumor‐bearing mice were recorded. Histological analysis of heart, spleen, and kidney was conducted by staining these tissue sections with H&E solution.

### Statistical Analysis

The experiments were repeated at least three times. Mean ± standard deviation (SD) was shown in the data of some figures and the sample size (n) for each statistical analysis was shown in the figure caption. A two‐tailed unpaired t test was used to determine the statistical significance. GraphPad Prism 8 Software was used for the statistical analysis. For all tests, the statistical significance was indicated as *p < 0.05, **p < 0.01, and ***p < 0.001. The *p* < 0.05 was considered statistically significant.

## Conflict of Interest

The authors declare no conflict of interest.

## Supporting information

Supporting Information

## Data Availability

The data that support the findings of this study are available from the corresponding author upon reasonable request.
