# Supplementary material for: Dual‐Cascade Activatable Nanopotentiators Reshaping Adenosine Metabolism for Sono‐Chemodynamic‐Immunotherapy of Deep Tumors
Source: Adv Sci (Weinh). 2023 Feb 2;10(10):2207200. doi: 10.1002/advs.202207200 (PMC10074132; doi:10.1002/advs.202207200)
Supplement: Supplementary file 1 — Supporting Information [file ADVS-10-2207200-s001.pdf]

Dual-Cascade Activatable Nanopotentiators Reshaping Adenosine Metabolism for Sono-Chemodynamic-Immunotherapy of Deep Tumors

Meixiao Zhan, Fengshuo Wang, Yao Liu, Jianhui Zhou, Wei Zhao, Ligong Lu, Jingchao Li*, Xu He**

Dr. M. Zhan, Y. Liu, W. Zhao, Prof. L. Lu, Dr. X. He
Guangdong Provincial Key Laboratory of Tumor Interventional Diagnosis and Treatment, Zhuhai People's Hospital (Zhuhai hospital affiliated with Jinan University), Jinan University, Zhuhai, Guangdong 519000, China
E-mail: zhanmeixiao1987@126.com, hexu220@163.com

F. Wang, J. Zhou, Prof. J. Li
State Key Laboratory for Modification of Chemical Fibers and Polymer Materials, College of Biological Science and Medical Engineering, Donghua University, Shanghai 201620, China
Email: jcli@dhu.edu.cn

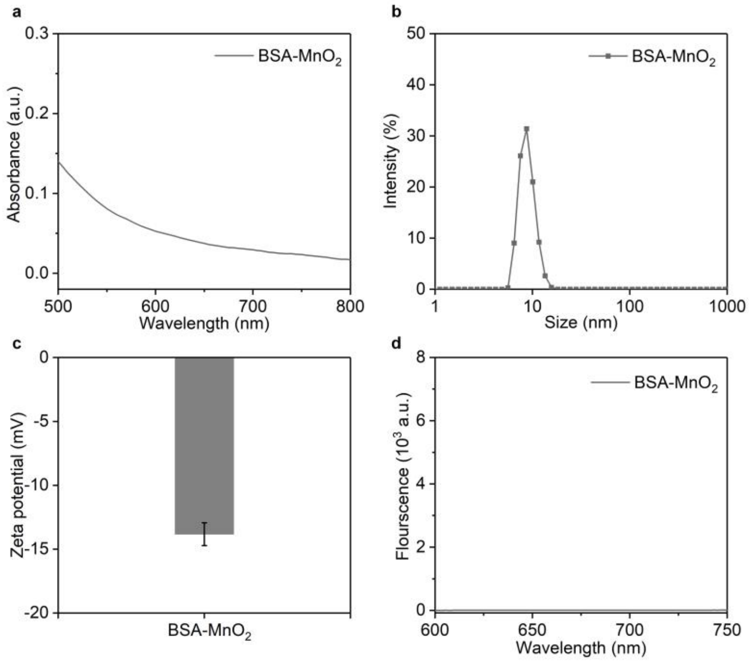

Figure S1. (a) UV-vis absorbance spectrum of BSA-MnO_2 nanoparticles. (b) Hydrodynamic size of BSA-MnO_2 nanoparticles. (c) Zeta potential of BSA-MnO_2 nanoparticles (n = 3). (d) Fluorescence spectrum of BSA-MnO_2 nanoparticles. The data are presented as the means ± SDs.

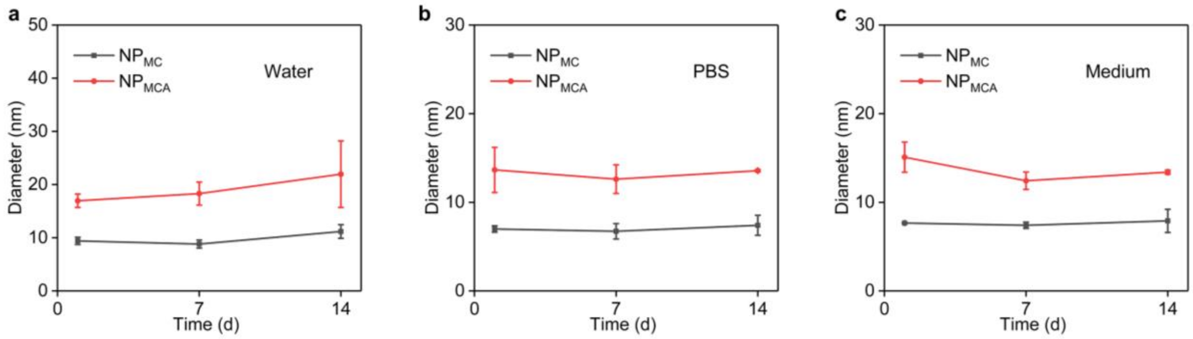

Figure S2. Hydrodynamic size of NP_{MC} and NP_{MCA} measured in (a) water, (b) PBS and (c) cell culture medium. The data are presented as the means ± SDs (n = 3).

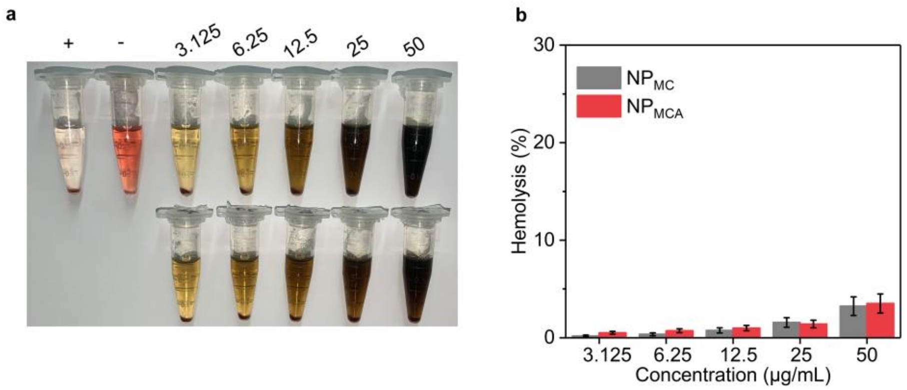

Figure S3. (a) Photographs of red blood cells after different treatments and centrifugation. (b) Hemolysis ratio of red blood cells in different treatment groups. The data are presented as the means ± SDs (n = 3).

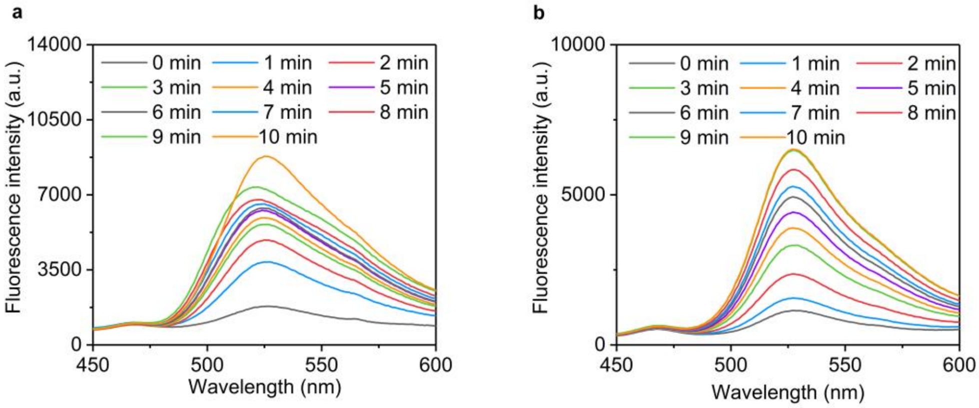

Figure S4. (a) Fluorescence spectra of SOSG in solution containing NP_{MC} after US treatment for different time. (b) Fluorescence spectra of SOSG in solution containing NP_{MCA} after US treatment for different time.

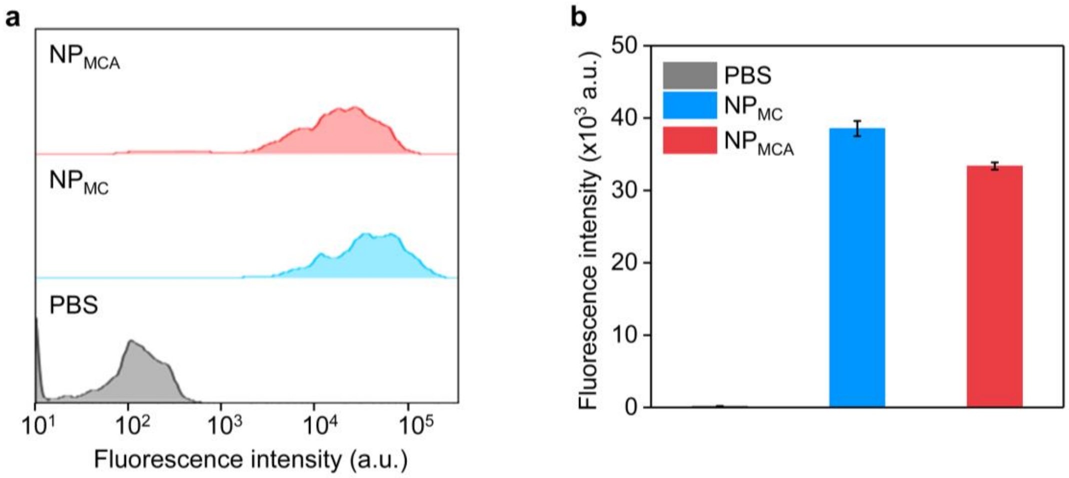

Figure S5. (a) Flow cytometry analysis of cellular uptake of NP_{MC} and NP_{MCA} by cancer cells. (b) Fluorescence intenisty of cancer cells after different treatments. The data are presented as the means ± SDs (n = 3).

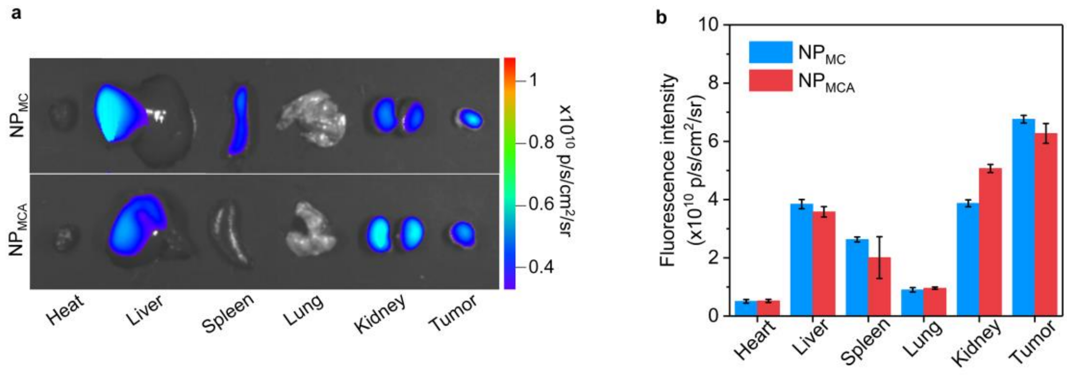

Figure S6. (a) Fluorescence images of tumor, liver, kidney, lung, heart and spleen after intravenous injection of NP_{MC} and NP_{MCA}. (b) Fluorescence intensity of tumor, liver, kidney, lung, heart and spleen in different mice. The data are presented as the means ± SDs (n = 3).

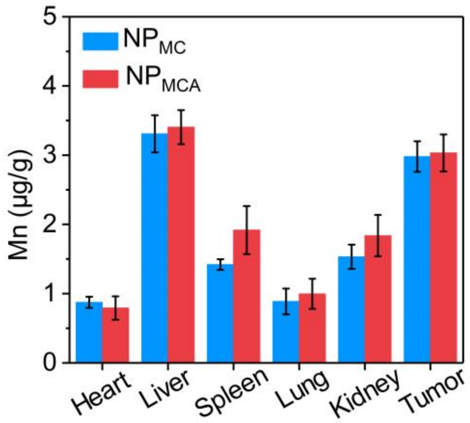

Figure S7. Quantitative analysis of Mn in different tissues after intravenous injection of NP_{MC} and NP_{MCA} into living mice. The data are presented as the means ± SDs (n = 5).

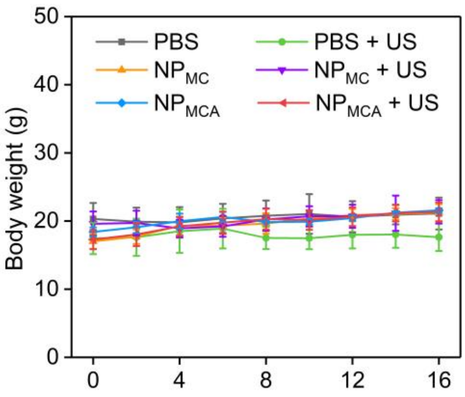

Figure S8. Body weight of 4T1 tumor-bearing mice after intravenous injection of NP$_{MC}$ and NP$_{MCA}$ with or without US irradiation at different time. The data are presented as the means ± SDs (n = 5).

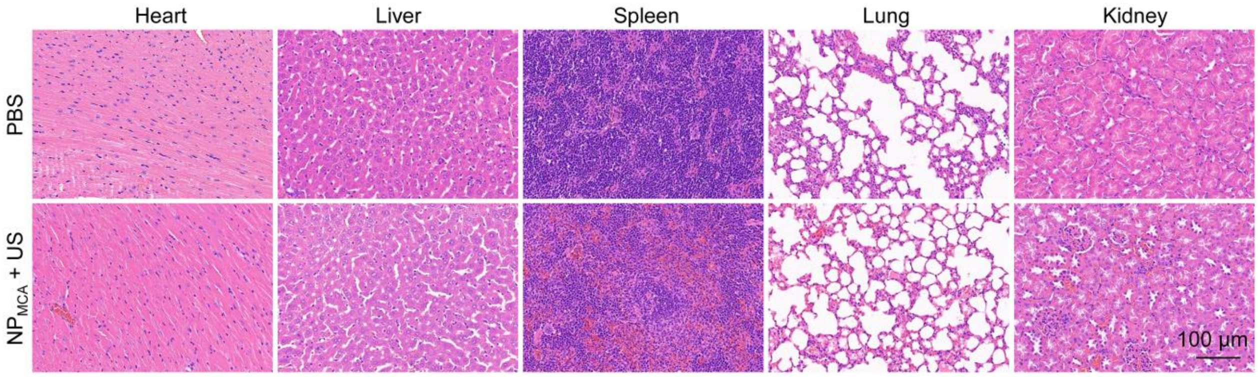

Figure S9. H&E staining images of heart, spleen, and kidney of mice after different treatments.
